# Oxo-M and 4-PPBP Delivery *via* Multi-Domain Peptide Hydrogel Toward Tendon Regeneration

**DOI:** 10.3389/fbioe.2022.773004

**Published:** 2022-01-27

**Authors:** Ga Young Park, Solaiman Tarafder, Samantha Lewis Eyen, Soomin Park, Ryunhyung Kim, Zain Siddiqui, Vivek Kumar, Chang H. Lee

**Affiliations:** ^1^ Regenerative Engineering Laboratory, Center for Dental and Craniofacial Research, Columbia University Irving Medical Center, New York, NY, United States; ^2^ Department of Bio-Medical Engineering, New Jersey Institute of Technology, Hoboken, NJ, United States

**Keywords:** tendon regeneration, small molecules, tendon stem/progenitor cells, multi-domain peptide, controlled delivery

## Abstract

We have recently identified novel small molecules, Oxo-M and 4-PPBP, which specifically stimulate endogenous tendon stem/progenitor cells (TSCs), leading to potential regenerative healing of fully transected tendons. Here, we investigated an injectable, multidomain peptide (MDP) hydrogel providing controlled delivery of the small molecules for regenerative tendon healing. We investigated the release kinetics of Oxo-M and 4-PPBP from MDP hydrogels and the effect of MDP-released small molecules on tenogenic differentiation of TSCs and *in vivo* tendon healing. *In vitro*, MDP showed a sustained release of Oxo-M and 4-PPBP and a slower degradation than fibrin. In addition, tenogenic gene expression was significantly increased in TSC with MDP-released Oxo-M and 4-PPBP as compared to the fibrin-released. *In* *vivo*, MDP releasing Oxo-M and 4-PPBP significantly improved tendon healing, likely associated with prolonged effects of Oxo-M and 4-PPBP on suppression of M1 macrophages and promotion of M2 macrophages. Comprehensive analyses including histomorphology, digital image processing, and modulus mapping with nanoindentation consistently suggested that Oxo-M and 4-PPBP delivered via MDP further improved tendon healing as compared to fibrin-based delivery. In conclusion, MDP delivered with Oxo-M and 4-PPBP may serve as an efficient regenerative therapeutic for *in situ* tendon regeneration and healing.

## Introduction

Tendons are dense fibrous tissues with the primary function of transferring mechanical forces from muscle to bone. Injuries to tendons can be caused by laceration, contusion, or tensile overload, which account for 50% of all musculoskeletal injuries in the US (Tozer and Duprez, 2005; Spindler et al., 2006; Fleming et al., 2009; Chen et al., 2013; Voleti et al., 2012). For example, rotator cuff injuries affect over 30% of Americans over 60 years of age, leading to over 50,000 surgical repairs annually (Milgrom et al., 1995; Tempelhof et al., 1999; Chen et al., 2009). In addition, approximately 11% of runners in the US suffer from Achilles tendinopathy (Chen et al., 2009), and there are 5 million new cases of tennis elbow (lateral epicondylitis) each year (Chen et al., 2009). This results in an enormous healthcare burden, with treatment for tendon injuries exceeding $30 billion per year in the US alone (Chen et al., 2009; Kew et al., 2011). Injuries to adult tendons do not spontaneously heal and frequently end up with scar-like tissue—exhibiting high cellularity, disarrayed collagen fibers, and poor mechanical properties (Thomopoulos et al., 2003; Voleti et al., 2012).

To improve tendon healing, various cell types including tenocytes, dermal fibroblasts, and stem/progenitor cells have been applied in tendon tissue engineering *in vitro* or in animal models (Juncosa-Melvin et al., 2005; Juncosa-Melvin et al., 2006a; Juncosa-Melvin et al., 2006b; Ouyang et al., 2006; Juncosa-Melvin et al., 2007; Fan et al., 2008; Nirmalanandhan et al., 2008; Fan et al., 2009; Hsu et al., 2010; Nourissat et al., 2010; Kew et al., 2011; Devana et al., 2018; Goncalves et al., 2018; Shen et al., 2018). Promising progress has been made in stem cell-based tendon regeneration *in vitro* and in animal models, despite the lack of clinical availability (Fan et al., 2008; Fan et al., 2009; Butler et al., 2010). Recently, we devised a novel *in situ* tissue engineering approach for tendon regeneration by activating endogenous stem/progenitor cells (Lee et al., 2015). We have identified perivascular-originating TSCs capable of guiding regenerative healing of tendons when stimulated by a connective tissue growth factor (CTGF) (Lee et al., 2015). Further investigation into molecular mechanisms of action led us to discover a combination of small molecules, Oxo-M and 4-PPBP, sharing intracellular signaling with CTGF, promoting tendon healing by harnessing endogenous TSCs (Tarafder et al., 2019). In addition, our data suggest that Oxo-M and 4-PPBP specifically target CD146^+^ TSCs via muscarinic acetylene receptors (AChRs) and sigma 1 receptor (σ1R) pathways (Tarafder et al., 2019). Given that there is no need for cell isolation, culture expansion, and transplantation, *in situ* tendon regeneration by delivery of Oxo-M and 4-PPBP has significant translational potential (Li et al., 2013).

Despite several advantages (Li et al., 2013), small molecule-based regenerative therapies have several limitations. A major outstanding challenge is the fast release of small molecules, likely linked with reduced bioactivity *in vivo* (Tarafder et al., 2019). This may serve as a significant roadblock in developing Oxo-M and 4-PPBP as regenerative therapeutics applicable in large, pre-clinical animal models and humans for which tendon healing likely takes longer than tendon healing in small animal models (Hast et al., 2014). Previously, we investigated the efficacy of controlled delivery of Oxo-M and 4-PPBP via poly (lactic-co-glycolic acids) (PLGA) microspheres (µS) (Tarafder et al., 2019). Sustained release of Oxo-M and 4-PPBP from PLGA µS resulted in a significant enhancement in tendon healing (Tarafder et al., 2019). However, degradation byproducts of PLGA potentially lower local pH, possibly leading to inflammation and disrupted tissue healing (Fu et al., 2000; Thackaberry et al., 2017). Accordingly, a biocompatible, reliable, injectable, and safe vehicle for controlled release of Oxo-M and 4-PPBP is required for facile translation.

In this study, we applied an injectable and self-assembling multidomain peptide (MDP) hydrogel (Kumar et al., 2015a; Kumar et al., 2015b) for controlled delivery of Oxo-M and 4-PPBP. The MDP hydrogel is composed of the sequence KKSLSLSLRGSLSLSLKK (termed K2). MDP self-assembles into *β*-sheets that further form entangled fibrous meshes (Kumar et al., 2015a; Kumar et al., 2015b). These highly hydrated meshes generate nanofibrous hydrogels that can be tuned to promote controlled delivery of various bioactive cues (Kumar et al., 2015a; Kumar et al., 2015b). Our previous studies confirmed the biocompatibility and non-acidic degradation products of MDP (Kumar et al., 2015a; Kumar et al., 2015b). Here, we investigated the efficacy of MDP hydrogel with sustained release of Oxo-M and 4-PPBP both *in vitro* and *in vivo* regarding tenogenic differentiation of TSCs, macrophage polarization, and tendon healing. The outcomes of MDP regarding the sustained release of Oxo-M and 4-PPBP and consequent regenerative tendon healing were quantitatively compared with fibrin gel, the simple carrier for the small molecules tested in our previous work (Tarafder et al., 2019).

## Materials and Methods

### Isolation and Sorting of CD146^+^ TSC

CD146^+^ TSCs were isolated from patella tendons (PT) of 12-week-old Sprague-Dawley (SD) rats, as per our prior methods (Lee et al., 2015). Briefly, the harvested PT was cleaned, minced, and then digested with 2 mg/ml collagenase at 37°C for 4 h. After centrifugation of the digest, the pellet was re-suspended in Dulbecco’s Modified Eagle Medium-Low Glucose (DMEM-LG; Sigma, St. Louis, MO) containing 10% fetal bovine serum (FBS; Gibco, Invitrogen, Carlsbad, CA) and 1% penicillin-streptomycin antibiotic (Gibco, Invitrogen, Carlsbad, CA). Then CD146^+^ cells were sorted using a magnetic cell separation kit (EasySep™, StemCells™ Technologies, Cambridge, MA).

### MDP Hydrogel for Controlled Delivery of Oxo-M and 4-PPBP

MDP were designed based on previously published sequences: SL: K_2_(SL)_6_K_2_ (Kumar et al., 2015b). All peptides, resin, and coupling reagents were purchased from CEM (Charlotte, NC). Standard solid phase peptide synthesis was performed on a CEM Microwave peptide synthesizer using Rinkamide resin with 0.37 mM loading, with C-terminal amidation and N-terminal acetylation. After cleavage from resin, peptides were dialyzed with 500–1200 MWCO dialysis tubing (Sigma-Aldrich, St. Louis, MO) against DI water. Peptides were subsequently lyophilized, confirmed for purity using electron-spray ionization mass spectrometry using MicroTOF ESI (Bruker Instruments, Billerica, MA), and reconstituted at 20 mg/ml (20 wt%) in sterile 298 mM sucrose. Gelation of the peptide was achieved by the addition of volume equivalents of pH 7.4 buffer with PBS or HBSS. Then Oxo-M (10 mM) and 4-PPBP (100 µM) were loaded at 10–50 µL in 1 ml of MDP. *In vitro* release profiles were measured by incubating 1 ml of MDP hydrogel encapsulated with Oxo-M or 4-PPBP in PBS or 0.1% BSA at 37°C with gentle agitation. The samples were centrifuged at the selected time points, followed by measuring concentrations in the supernatants with a UV-Vis spectroscope (Nanodrop™ 2000, ThermoFisher Scientific, Waltham, MA) at 230 and 207 nm wavelengths for Oxo-M and 4-PPBP, respectively. A shear recovery test was conducted to characterize the viscoelastic properties of the hydrogel as per our prior method (Nguyen et al., 2018). Briefly, 40 μL of the samples (MDP and MDP + OP) were pipetted underneath a 4 mm geometry with a gap of 250 μm using a Malvern Kinexus Ultra^+^ rheometer (NETZSCH North America, Burlington, MA). Repeated strains (1% strain/1 Hz for 5 min, followed up with 100% strain/1 Hz for 1 minute) were applied and dynamic storage moduli were measured.

### MDP Degradation

In the *in vitro* degradation test, MDP hydrogel was prepared with and without Oxo-M and 4-PPBP, as labeled with Alexa Fluor^®^ 488 dye. Fibrin gel (50 mg/ml fibrinogen and 50 U/ml thrombin) with and without Oxo-M and 4-PPBP was prepared as a comparison group. The final concentrations of Oxo-M and PPBP in fibrin gel and MDP were 1 mM and 10 μM, respectively. Then an equal volume (80 µL) of each gel (N = 3) was placed into the wells of a 24-well plate and kept in PBS for the duration of the study. At pre-determined time points, fluorescent images of the samples were taken using the Maestro™ *in vivo* fluorescence imaging system (Cambridge Research & Instrumentation, Inc., Woburn, MA, United States). The images were processed by ImageJ to calculate percent degradation from the area of the remaining gels.

### *In vitro* Assessment of the Efficacy of Sustain-Released Oxo-M and 4-PPBP

The efficacy of sustained release of Oxo-M and 4-PPBP from MDP hydrogel was tested for TSCs differentiation with transwell co-culture. Briefly, MDP encapsulated with Oxo-M and 4-PPBP was applied to Transwell^®^ inserts with a 0.4 µm pore membrane, where TSCs (80–90% confluence) were cultured in the bottom wells. This co-culture model allows the released small molecules to be transported while preventing direct contact between cells and MDP. At 1 week of culture with tenogenic induction supplements (Lee et al., 2015), mRNA expressions of tendon-related genes including collagen type I and III (COL-I and III), tenascin-C (Tn-C), vimentin (VIM), tenomodulin (TnmD), fibronectin (Fn) and scleraxis (Scx) were measured by quantitative RT-PCR using the Taqman™ gene expression assay (Life Technologies; Grand Island, NY) as per our established protocols (Lee et al., 2015). The quantitative measures for tenogenic differentiation of TSCs by control-delivered Oxo-M and 4-PPBP were compared with the release from fibrin gel (50 mg/ml fibrinogen +50 U/ml thrombin).

### *In Vivo* Tendon Healing by Controlled Delivery of Oxo-M and 4-PPBP

As per prior work, MDP-encapsulated with Oxo-M and 4-PPBP was delivered into a fully transected rat patellar tendon (PT) (Lee et al., 2015). All animal procedures followed an IACUC approved protocol, and 12-week-old Sprague-Dawley (SD) rats (*n* = 4 per group and time point) were used. Upon anesthesia, a 10 mm longitudinal incision was made just medial to the knee. After exposing PT, a full-thickness transverse incision was made using a no. 11 blade scalpel. MDP hydrogel was applied on the transection site with or without Oxo-M and 4-PPBP. After creating a bone tunnel at the proximal tibia using a 0.5 mm drill, a 2-0 Ethibond suture (Ethicon Inc., Somerville, NJ, United States) was passed through the tibial tunnel and quadriceps in a cerclage technique. The surgical site was then closed using 4.0 absorbable (continuous stitch) for the subcutaneous layer and 4.0 PDS and monocryl (interrupted stitches) for the skin closure. At 2 weeks post-op, the animals were euthanized. The quality of tendon healing associated with endogenous TSCs was analyzed using H and E, Picrosirius-red (PR) polarized imaging, and automated quantitative imaging analysis for collagen fiber orientation. To image whole tissue sections containing any spatial features, slide scanning was performed using the Aperio AT2 scanner (Leica Biosystems Inc. Buffalo Grove, IL). From H and E stained tissue sections (*n* = 10 per group), the quality of tendon healing was quantitatively evaluated using a modified Watkins scoring system (Loppini et al., 2015), covering cellularity, vascularity, cell alignment, amount and size of collagen fibers, and wave formation. In addition, immunofluorescence (IF) was performed for macrophage polarization markers, including inducible nitric oxide synthase (iNOS) (PA1-036, Thermo Fisher), and CD163 (NMP2-39099, Novus Biologicals), as co-labeled with DAPI. Anti-inflammatory cytokine IL-10 (AF519-SP, Novus Biologicals) and tissue inhibitor of metalloproteinases-3 (TIMP-3) (ab39184, Abcam) were also evaluated using IF (*n* = 4 biological replicates; *n* = 5 sections per biological sample). The labeled tissue sections were imaged using the Aperio AT2 scanner with fluorescence. From randomly selected slides (*n* = 15 per group), total cell number of cells and blood vessels per unit area, or quantification of cell density and blood vessels, were calculated using ImageJ as per our well-established protocol (Lee et al., 2015; Tarafder et al., 2019).

### Automated Image Analysis for Collagen Alignment

As per our well-established methods (Lee et al., 2005; Lee et al., 2015; Tarafder et al., 2019), we analyzed the collagen fiber orientation in PR stained tissue sections using a digital image processing technique. The automated image-processing method calculated the local directionality and angular deviation (AD) in circularly polarized PR-stained images. The analysis of each image yielded a distribution of fiber orientations, ranging from -90° to 90°, where 0° was defined as the vertical direction. The degree of collagen fiber alignment was quantified using the AD. The value of the AD was calculated using circular statistics (Lee et al., 2005; Lee et al., 2015) implemented with MATLAB (Mathworks Inc., Natick, MA, United States). For the digital imaging processing, a total of 15 different image samples were used per group.

### Modulus Mapping With Nanoindentation

To assess the maturation and homogeneity of extracellular matrix (ECM) in the healing zone, we performed modulus mapping with nanoindentation on the tendon section as per well-established methods (Akhtar et al., 2009). Briefly, the nanoindentation was conducted using a PIUMA™ nano-indenter (Optics11, Amsterdam, Netherlands) with a 1 μm probe. The unfixed and unstained tissue sections were mounted on the embedded high-precision mobile X-Y stage and a maximum force of 10 mN was applied at every 20 μm distance from the original defect site to determine the effective indentation modulus (E_Eff_) across a healed region over a selected 400 μm × 400 µm area. The measured E_Eff_ values were displayed in the XYZ plane to visualize their homogeneity over the unit area. Then the E_Eff_ values from control, fibrin with Oxo-M and 4-PPBP (Fib + OP), and MDP + OP groups were normalized to those of the intact region in the corresponding tendon samples.

### Effect of Oxo-M and 4-PPBP on Macrophage Polarization

Given the essential roles of macrophages during tendon healing (Sunwoo et al., 2020), we evaluated the effects of Oxo-M and 4-PPBP on macrophage polarization *in vitro*. Briefly, THP-1 human monocytes (ATCC^®^, Manassas, VA) were cultured in complete RPMI media (ThermoFisher Scientific, Waltham, MA), supplemented with 10% heat-inactivated FBS and 1% penicillin/streptomycin (Witherel et al., 2018). For differentiation of THP-1 monocytes into un-activated (M0) macrophages, phorbol 12-myristate 13-acetate (PMA) was applied at 320 nM for 16 h. For M1 polarization, 100 ng/ml of lipopolysaccharide (LPS) and 100 ng/ml of recombinant human interferon-γ (IFN-γ) were applied for 48 h. For M2 polarization, 40 ng/ml of recombinant human interleukin-4 (IL-4) and 20 ng/ml of recombinant human IL-13 were applied as per well-established protocols (Witherel et al., 2018). Oxo-M (1 mM) and 4-PPBP (10 µM) were applied along with the M1 and M2 polarization stimuli. After 48 h, all cells were detached by gentle scraping, followed by RNA isolation for qRT-PCR analysis for M1 and M2 polarization mRNA markers, including tumor necrosis factor-alpha (TNF-α), IL-1β, mannose receptor C-type 1 (MRC1), and platelet-derived growth factor b (PDGFb).

### Statistical Analysis

For all the quantitative data, following confirmation of normal data distribution, one-way analysis of variance (ANOVA) with post-hoc Tukey HSD tests was used with a *p* value of 0.05. Sample sizes for all quantitative data were determined by power analysis with one-way ANOVA using a level of 0.05, power of 0.8, and an effect size of 1.50 chosen to assess matrix synthesis, gene expressions, and structural properties in the regenerated tendon tissues and controls.

## Results

### Sustained Release of Oxo-M and 4-PPBP From MDP Hydrogel Promotes Tenogenic Differentiation

**FIGURE 1:**
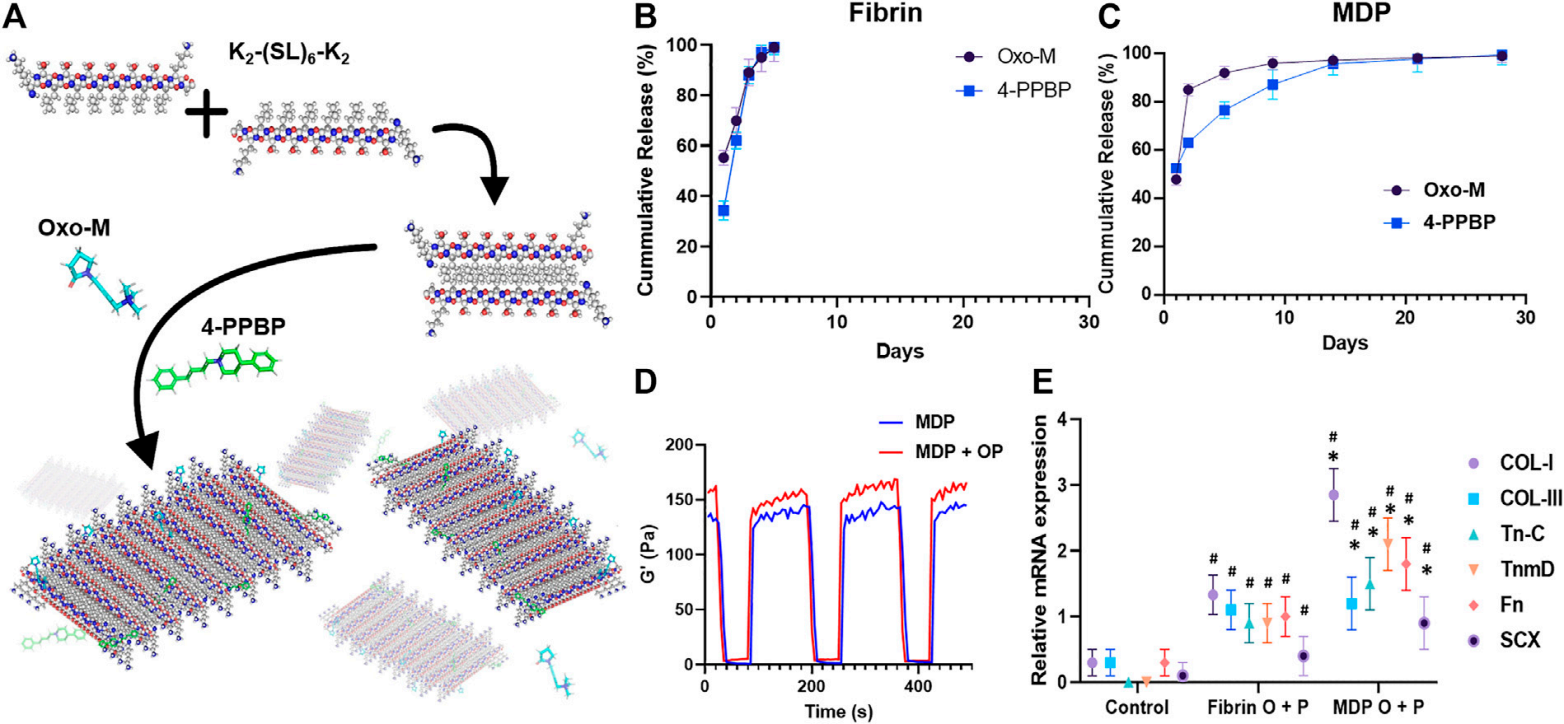
Multidomain peptide (MDP) hydrogel as a controlled delivery vehicle for Oxo-M and 4-PPBP. MDP self-assembles supra-molecularly into nanofibers that encapsulate drugs while maintaining shear thinning and shear recovery properties **(A)**. This allows for facile aspiration and delivery as depots into tissue sites for the localized release of small molecule drugs from biodegradable peptide scaffolds. Oxo-M and 4-PPBP loaded in fibrin gel were fully released by 3,4 days **(B)**, whereas they showed sustained release from MDP hydrogel up to 25 days **(C)**. Tenogenic gene expressions were significantly higher in TSCs cultured under Transwell^®^ inserts with fibrin and MDP hydrogel releasing Oxo-M and 4-PPBP **(C)**. Dynamic storage moduli of MDP and MDP + OP hydrogels under repeated strain cycles (1% strain and 100% strain), showing a recovery of viscoelastic properties within 10 s even after repeated strain cycles **(D)**. Oxo-M and 4-PPBP release from MDP hydrogel resulted in significantly higher gene expressions as compared to what was released from fibrin **(E)** (n = 5 per group; *: p < 0.001 compared to fibrin group; ^#^: p < 0.001 compared to control).

*In vitro* release kinetics showed that Oxo-M and 4-PPBP are fully released from fibrin within 3, 4 days (Figure 1B). However, Oxo-M and 4-PPBP showed sustained release from MDP up to 14–25 days (Figure 1C). Dynamic storage moduli under the shear recovery test showed the resilience of both for MDP and MDP + OP with a quick recovery from repeated strains (1–100% 1Hz) (Figure 1D). MDP + OP showed a higher peak shear storage modulus than MDP, without statistical significance (*n* = 5 per sample). Expressions of tendon-related genes, including COL-I and III, Tn-C, TnmD, Fn, and Scx, were significantly increased in TSCs cultured under a Trans-well insert loaded with Oxo-M and 4-PPBP in fibrin or MDP hydrogel, in comparison with control with no treatment by 1 week (Figure 1D) (*n* = 5 per group; *p* < 0.001). In addition, all the tested tenogenic gene expressions were significantly higher in MDP + OP than in Fib + OP (Figure 1E) (*n* = 5 per group; *p* < 0.001), suggesting a positive effect of the prolonged-release from MDP hydrogel.

### *In vitro* Degradation

**FIGURE 2:**
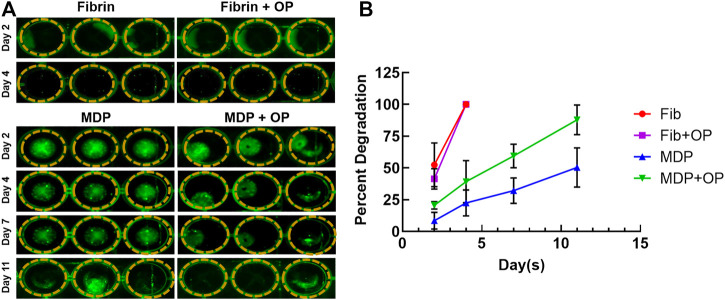
In vitro degradation of MDP and fibrin gel with and without Oxo-M and 4-PPBP. Fluorescence-labeled fibrin and MDP with or without Oxo-M and 4-PPBP (OP) were imaged **(A)**, and the integral of signal intensities were quantified **(B)** (*n* = 3 per group). MDP delivered with Oxo-M and 4-PPBP enhanced tendon healing *in vivo*.

Images of fluorescence-labeled hydrogels showed the remaining amounts of fibrin and MDP hydrogels throughout *in vitro* degradation (Figure 2A). Fibrin appeared to fully degrade by 4 days *in vitro* with and without Oxo-M and 4-PPBP. In contrast, MDP hydrogel showed muted degradation by 11 days *in vitro* (Figure 2A). Quantitative fluorescence signal strength measured by the Maestro™ imaging system consistently showed that MPD showed ∼48% volumetric reduction by 11 days, which is significantly slower than fibrin gel, which showed a 100% degradation by 4 days (Figure 2B). In addition, delivering Oxo-M and 4-PPBP in the MDP hydrogel significantly accelerated the *in vitro* degradation (Figure 2B).

Oxo-M and 4-PPBP release from MDP hydrogel resulted in significantly higher gene expressions as compared to what was released from fibrin (**E**) (*n* = 5 per group; *: *p* < 0.001 compared to fibrin group; #:*p* < 0.001 compared to control).

**FIGURE 3:**
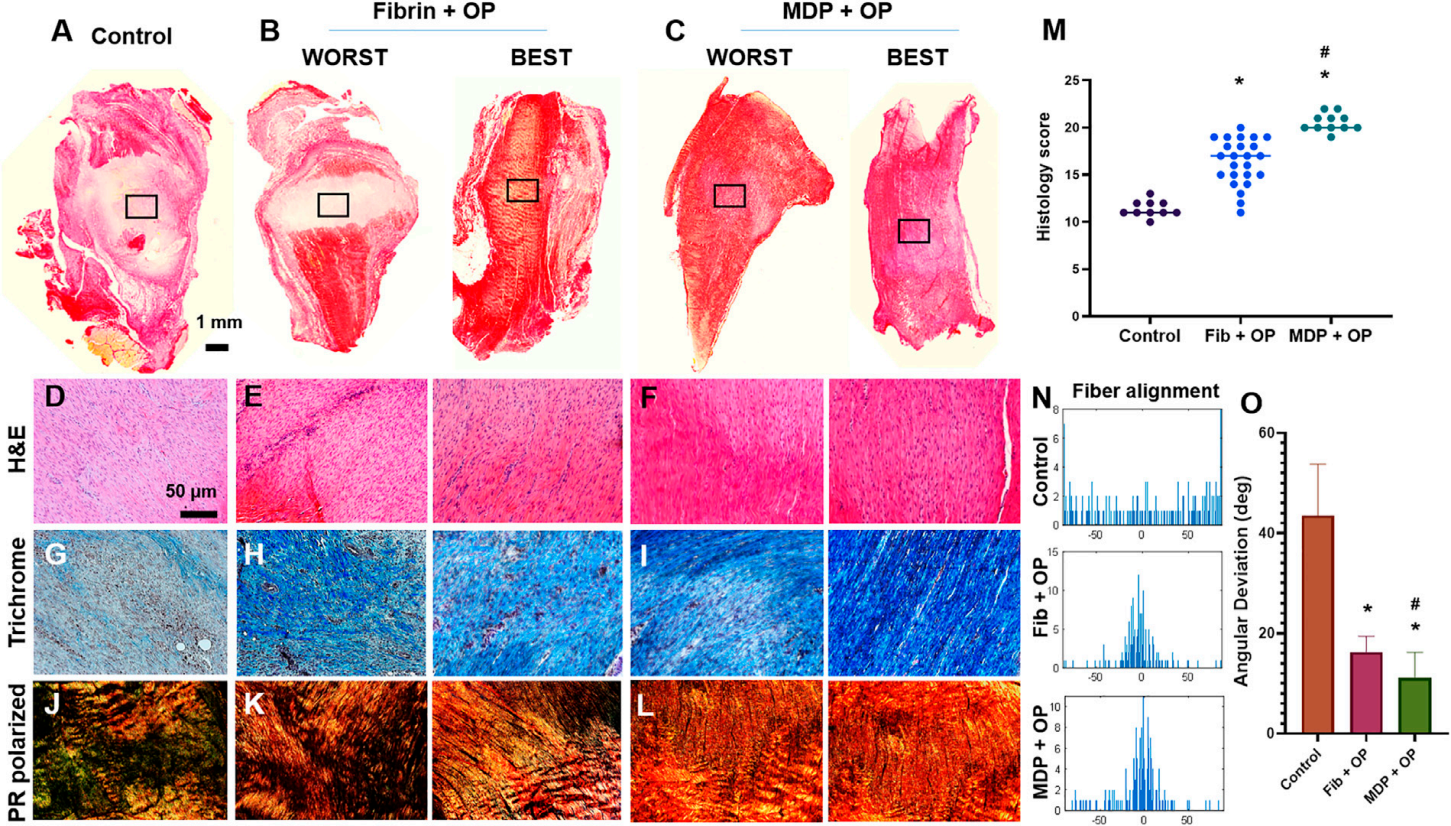
*In vivo* tendon healing by 2 weeks. The control ended up with scar-like healing with disrupted matrix and high cellularity **(A–D)**. In contrast, fibrin and MDP delivered with Oxo-M and 4-PPBP showed notable improvement in tendon healing **(B–F)**. Masson’s trichrome showed higher collagen deposition in the healing zone with Oxo-M and 4-PPBP delivery via fibrin and MDP **(G–I)**. Polarized PR images showed higher collagen orientation with MDP + OP than fibrin + OP **(J,K)**. There were some variances in the healing outcome with fibrin + OP **(B–K)** compared to a more consistent outcome with MDP + OP **(C–L)**. Quantitatively, MDP + OP resulted in significantly higher histological scores with a relatively small variance as compared to Fib + OP **(M)** (*n* = 10–25 per sample; *: *p* < 0.001 compared to control; *p* < 0.001 compared to Fib + OP). Digital imaging processing shows a narrow distribution of fiber orientation angels in MDP + OP and Fib + OP compared to the spread histogram in control **(N)**. Quantitative angular deviation (AD) value was significantly lower with MDP + OP as compared to fibrin + OP and control **(O)** (*:*p* < 0.0001 compared to control; #:*p* < 0.001 compared to Fib + OP; *n* = 10–15 per group). All images are representative best outcome for each group.

Fully transected rat PT without treatment ended up with scar-like healing with high cellularity, lacked collagen matrix, and disrupted collagen orientation by 2 weeks post-op (Figures 3A–J). In contrast, Oxo-M and 4-PPBP delivery via fibrin and MDP hydrogel significantly enhanced tendon healing with significantly improved structure (Figures 3B–E), dense collagen deposition (Figure 3H), and re-orientation of collagen fibers (Figure 3K) in comparison with control. A few tissue samples in the fibrin/Oxo-M and 4-PPBP (Fib + OP) groups showed somewhat suboptimal healing (Figure 3B), whereas MDP/Oxo-M and 4-PPBP (MDP + OP) resulted in a more consistent healing outcome (Figure 3C). Similarly, the collagen fibers appeared to be denser and better aligned in the MDP + OP group as compared to the Fib + OP group (Figures 3I–L). In addition, MDP + OP resulted in a significantly higher histological score with a smaller variance than Fib + OP with a larger variance (Figure 3M). Quantitatively, the cell density in control was significantly higher than that of Fib + OP and MDP + OP (Supplementary Figure S1) (*p* < 0.001). Similarly, the number of blood vessels was significantly higher in control as compared to those of Fib + OP and MDP + OP (Supplementary Figure S1) (*p* < 0.001). Consistently, digital imaging processing showed an improved collagen fiber orientation in MDP + OP and Fib + OP as compared to control (Figure 3N). The degree of collagen alignment quantified as AD value was superior with MDP + OP to Fib + OP (Figure 3O) (*n* = 15 per group; *p* < 0.001).

### MDP + OP Improved Magnitude and Distribution of Indentation Moduli

**FIGURE 4:**
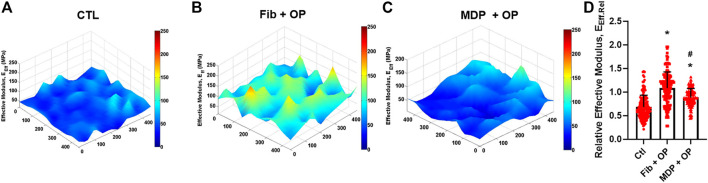
Modulus mapping by nanoindentation of tendon sections **(A–C)**, showing more homogenous distribution of indentation moduli with MDP + OP as compared to Fib + OP. Relative effective modulus (E_Eff_.Rel) at healing zone in respect to corresponding intact area **(D)** were significantly higher in Fib + OP and MDP + OP than control. E_Eff_.Rel showed larger variance in Fib + OP than MDP + OP (n = 100–150 per group; *:*p* < 0.0001 compared to control; #:*p* < 0.0001 compared to Fib + OP). Effect of Oxo-M and 4-PPBP on macrophage polarization.

Modulus mapping with nanoindentation displayed the distributions of effective indentation modulus (E_Eff_) over 400 μm × 400 µm areas in the healing regions (Figures 4A–C). The control group showed lower E_Eff_ values with a somewhat homogeneous distribution (Figure 4A). Fib + OP showed higher E_Eff_ values with a less homogenous distribution (Figure 4B), and MDP + OP showed a highly homogenous distribution (Figure 4C). Quantitatively, the control group showed a lower average E_Eff_ at the healing zone than the intact tendon, whereas Fib + OP showed an average E_Eff_ significantly higher than the control (Figure 4D) (*n* = 100–150 per group; *p* < 0.0001). MDP + OP showed E_Eff_ at a similar level with intact tendons (Figure 4D) (*n* = 100–150 per group; *p* < 0.0001). Consistently with the E_Eff_ distribution (Figures 4A–C), MDP + OP showed a smaller variance in E_Eff_ than Fib + OP (Figure 4D).

**FIGURE 5:**
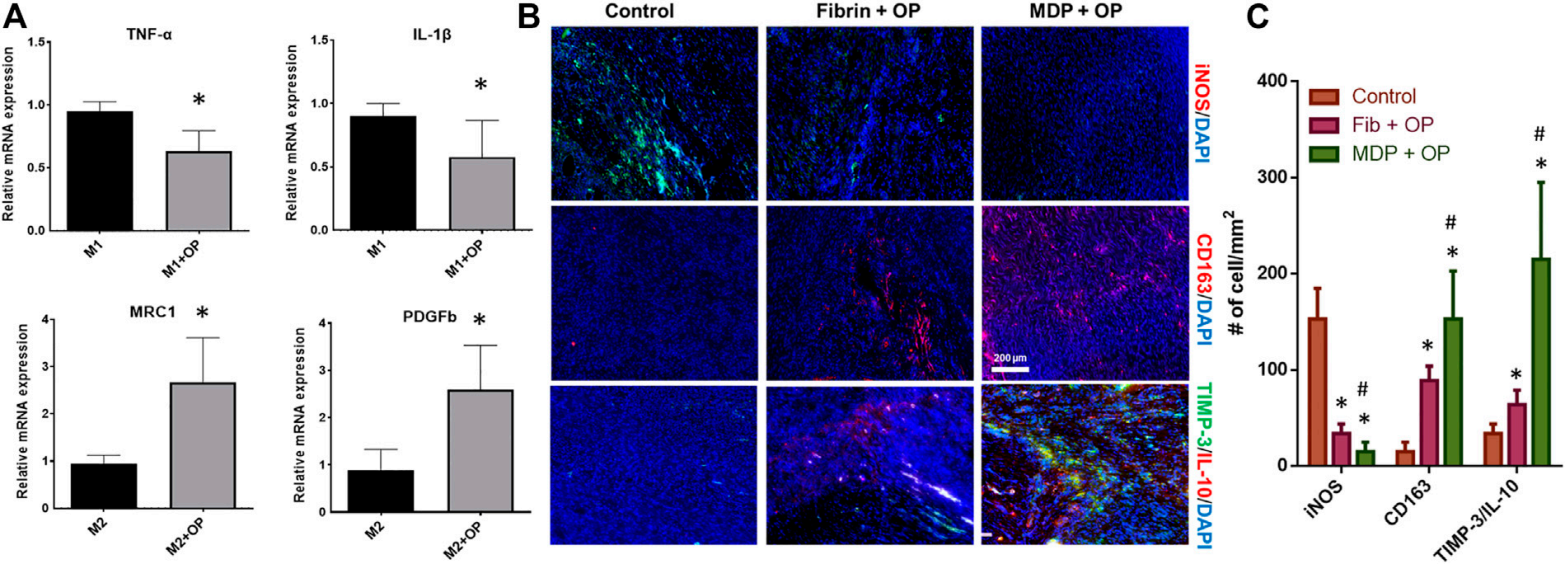
Effect of Oxo-M and 4-PPBP on macrophage polarization **(A)** (*:*p* < 0.001 compared control). Immunofluorescence of macrophage and anti-inflammatory markers **(B)**. Quantitative analysis of cell numbers **(C)** showed a significantly lower number of iNOS + M1-like macrophages OP delivery. MDP + OP showed an increased number of CD163 + M2-like cells as compared to fibrin + OP. TIMP-3 and IL-10 showed robust expression in MDP + OP group in comparison with Fib + OP (*n* = 15 per group; *:*p* < 0.001 compared to control; #:*p* < 0.001 compared to Fib + OP).

By 48 h of M1 polarization of THP-1 derived macrophages induced by LPS and IFN-γ, the treatment with OP significantly reduced mRNA expressions of TNF-α and IL-1β (Figure 5A). In contrast, Oxo-M and 4-PPBP significantly promoted M2 polarization induced by IL-4 and IL-10, with elevated levels of MRC1 and PDGFb (Figure 5A) (*n* = 6 per group; *:*p* < 0.001). *In vivo*, OP delivery via fibrin and MDP resulted in a significantly lower number of iNOS^+^ M1-like cells by 2 weeks post-op (Figure 5B). The number of CD163^+^ M2-like macrophages was significantly increased with OP delivery (Figure 5B). In addition, MDP + OP showed more M2-like CD163^+^ cells as compared to Fib + OP (Figure 5B). High magnification images of the immunofluorescence of MDP + OP samples and control images without primary antibodies are provided in Supplementary Figure S2.

## Discussion

Our findings suggest an effective and reliable approach to enable the controlled delivery of small molecules that improve regenerative tendon healing by harnessing endogenous stem/progenitor cells. The unique chemical characteristics of MDP hydrogels, self-assembling into *β*-sheets, enable the entrapment of small molecular weight drugs such as Oxo-M and 4-PPBP, consequently providing sustained release over time. Given that MDP self-assembles through noncovalent interactions of alternating hydrophobic leucine residues and hydrogen bonding of hydrophilic serines (Kumar et al., 2015b), both hydrophilic Oxo-M and hydrophobic 4-PPBP were able to be loaded into MDP *β*-sheet and then showing sustained-release without notable difference in the release kinetics between Oxo-M and 4-PPBP (Figures 1B,C). In contrast to the previously used PLGA µS, MDP’s degradation byproducts do not change local pH, with good biocompatibility established in several prior studies (Kumar et al., 2015b). In addition, its unique near-instantaneous self-assembly in an aqueous solution allows drug solubilization and facile injection of MDP hydrogels into desired sites via a syringe needle, followed by near-instant *in situ* gelations (Kumar et al., 2015b). These characteristics further advocate the potential of MDP hydrogels as an efficient and controlled delivery vehicle.

A prolonged release of Oxo-M and 4-PPBP from MDP appeared to enhance the tenogenic differentiation of TSCs and modulate macrophages polarization. Our *in vitro* data suggests that Oxo-M and 4-PPBP may interfere with M1 polarization while promoting M2 polarization. Collective experimental evidence in several previous studies supports the temporal roles of inflammatory M1 macrophages and anti-inflammatory M2 macrophages in the early and late phases of tendon healing, respectively (Marsolais et al., 2001; Akhtar et al., 2009; Xu et al., 2020; Howell et al., 2021). Excessive or prolonged M1 macrophages are closely involved with inflammation and scarring, whereas M2 macrophages play essential roles in matrix synthesis and remodeling (Marsolais et al., 2001; Akhtar et al., 2009; Xu et al., 2020; Howell et al., 2021). Thus, prolonged activation of Oxo-M and 4-PPBP via controlled delivery with MDP may have promoted tendon healing by attenuating M1-mediated inflammation and M2-mediated anti-inflammatory cytokines and matrix remodeling. Consistently, we have observed elevated TIMP-3 and IL-10 with MDP + OP compared to Fib + OP by 2 weeks post-op.

The modulus mapping on sectioned tendon tissues by nanoindentation revealed interesting features in the healed ECM (Figure 4). The scar-like matrix formed in the control group showed a relatively homogenous distribution of indentation modulus with high moduli in an isolated area (Figure 4A). On the other hand, tendon tissue healed with OP delivered via fibrin gel increased the average indentation modulus but showed substantial inhomogeneity over the testing area (Figure 4B). Notably, tendons delivered with MDP + OP increased indentation moduli with a highly homogenous distribution (Figure 4C). These observations may suggest that a relatively inhomogeneous matrix in Fib + OP is likely due to the immaturity of the healed tendon matrix, and that a more mature tissue matrix in the MDP + OP group was formed by a prolonged release of OP leading to sustainedOP activation of M2 macrophages modulating inflammation and matrix remodeling.

Despite the promising outcomes, our study has several limitations, including the unknown *in vivo* degradation rate. Most biodegradable materials exhibit *in vivo* degradation rates markedly different from those observed in well-controlled *in vitro* studies (Tarafder et al., 2016; Nakagawa et al., 2019), likely associated with dynamic changes in the biochemical environment *in vivo* affected by inflammation, cell metabolism, and co-morbidities (Tarafder et al., 2016; Nakagawa et al., 2019). Thus, the actual degradation of MDP hydrogel and consequent release of Oxo-M and 4-PPBP may differ from the *in vitro* data. Nonetheless, such *in vivo* factors are speculated to affect the degradation of both fibrin and MDP, consequently validating our comparative *in vivo* study between the two different delivery vehicles. Fortunately, various state-of-the-art imaging modalities are being developed to track *in vivo* degradation and release via non-invasive measurements (Medintz et al., 2005; Han et al., 2015; Kim et al., 2020), which will likely serve as an efficient tool to optimize the delivery vehicles in follow-up studies further. Another limitation of this study is the missing tensile properties of *in vivo* tendon samples, primarily due to the small sample numbers. Although previous studies of ours and others have suggested a statistical correlation between the quantitative collagen orientation and tensile properties (Lake et al., 2010; Lee et al., 2015; Gutman et al., 2018; Tarafder et al., 2019), such an image-based assessment remains an incomplete functional evaluation, representing a limitation.

In conclusion, MDP may represent a highly efficient, injectable hydrogel system allowing controlled delivery of Oxo-M and 4-PPBP with a specific function to stimulate endogenous stem/progenitor cells and modulate macrophages toward tendon regeneration. Given that there is no need for cell translation, our approach with MDP releasing Oxo-M and 4-PPBP has a significant clinical impact as a highly translational approach to induce regenerative healing of tendons.

## Data Availability

The raw data supporting the conclusion of this article will be made available by the authors without undue reservation.
