# Supplementary material for: Oxo-M and 4-PPBP Delivery via Multi-Domain Peptide Hydrogel Toward Tendon Regeneration
Source: Front Bioeng Biotechnol. 2022 Jan 27;10:773004. doi: 10.3389/fbioe.2022.773004 (PMC8829701; doi:10.3389/fbioe.2022.773004)
Supplement: Supplementary file 1 [file DataSheet1.PDF]

Supplementary data

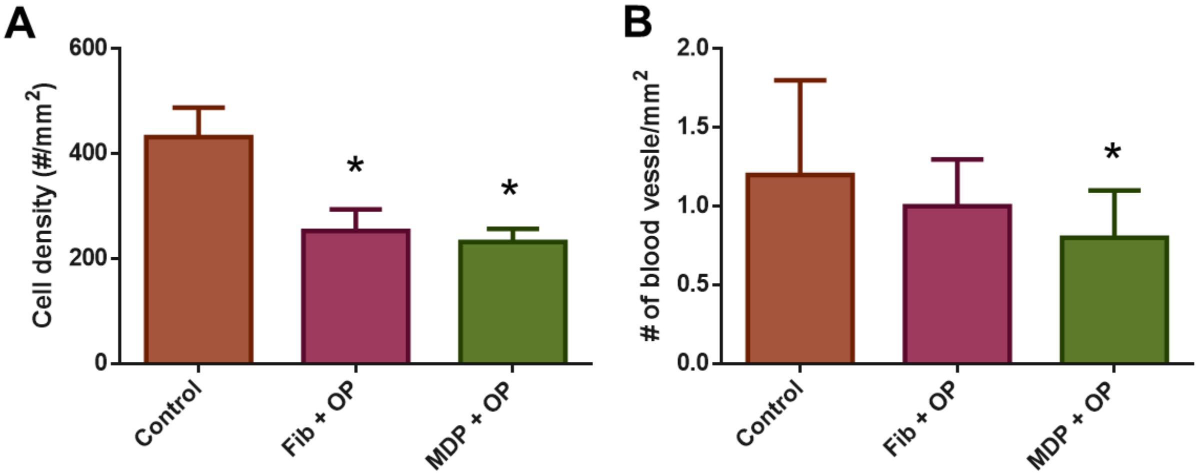

Supplementary Fig. 1. Quantitative analysis of cell density (**A**) and vascularization (**B**) at 2 wks post-op (*:p<0.001 compared to control; n = 15 per group).

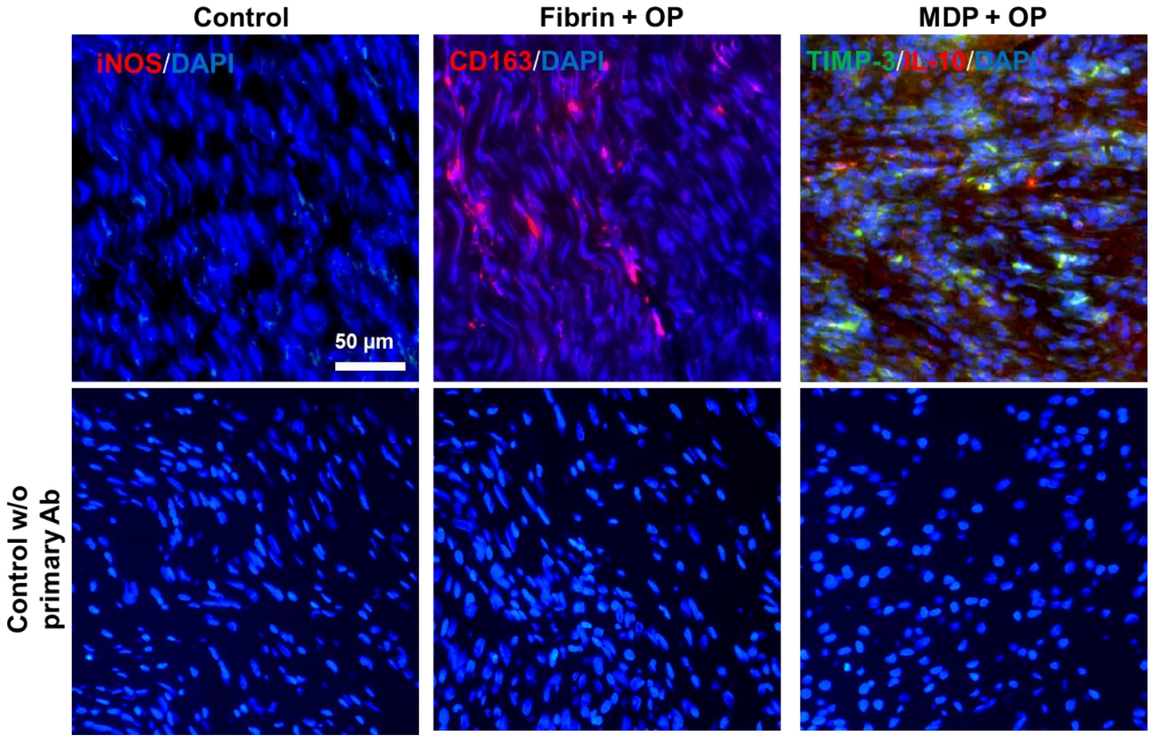

Supplementary Fig. 2. High magnification of iNOS, CD163 and TIMP-3/IL-10 immunostaining of tendon samples with MDP + OP, and negative control with no primary antibody.
